# Spontaneous rhythms in a harbor seal pup calls

**DOI:** 10.1186/s13104-017-3107-6

**Published:** 2018-01-03

**Authors:** Andrea Ravignani

**Affiliations:** 1 Research Department, Sealcentre Pieterburen, Hoofdstraat 94a, 9968 AG Pieterburen, The Netherlands; 2 Artificial Intelligence Lab, Vrije Universiteit Brussel, Pleinlaan 2, 1050 Brussels, Belgium; 3 Language and Cognition Department, Max Planck Institute for Psycholinguistics, Wundtlaan 1, 6525 XD Nijmegen, The Netherlands

**Keywords:** Bioacoustics, Rhythm, Vocal communication, Vocalization, Animal call, Marine mammal, Pinniped, Turn-taking, Timing, Evolution of speech, Harbor seal

## Abstract

**Objectives:**

Timing and rhythm (i.e. temporal structure) are crucial, though historically neglected, dimensions of animal communication. When investigating these in non-human animals, it is often difficult to balance experimental control and ecological validity. Here I present the first step of an attempt to balance the two, focusing on the timing of vocal rhythms in a harbor seal pup (*Phoca vitulina*). Collection of this data had a clear aim: To find spontaneous vocal rhythms in this individual in order to design individually-adapted and ecologically-relevant stimuli for a later playback experiment.

**Data description:**

The calls of one seal pup were recorded. The audio recordings were annotated using Praat, a free software to analyze vocalizations in humans and other animals. The annotated onsets and offsets of vocalizations were then imported in a Python script. The script extracted three types of timing information: the duration of calls, the intervals between calls’ onsets, and the intervals between calls’ maximum-intensity peaks. Based on the annotated data, available to download, I provide simple descriptive statistics for these temporal measures, and compare their distributions.

## Objective

Pinnipeds are a clade of marine mammals exhibiting a range of vocal behaviors [1, 2]. Testing rhythm and timing in pinnipeds is relevant to a number of cross-species evolutionary hypotheses relating rhythmic behaviors to vocal flexibility, social cognition, and brain plasticity [3–8].

Traditionally, the comparative study of rhythm and timing has spanned two main strands. Animal vocalizations and behaviors have been *recorded* in ecologically-relevant settings to unveil temporal structures. These observational approaches allowed little experimental control. Alternatively, animals have been *tested* in an operant setup, employing controlled external stimuli to trigger animals’ reactions. These other approaches traded ecological relevance for experimental control. While a few exceptions exist [9], animal research on rhythm and timing still needs to strike a good tradeoff between experimental rigor and ecological relevance.

Here I present the first step of an attempt to balance the two. I describe and share data on spontaneous vocal rhythms in a harbor seal pup. Audio recordings were collected with the explicit aim of finding the natural timing of vocal production in this individual, and design ecologically-relevant and individually-adapted stimuli for a future playback experiment [9–11].

After recording the animal, I annotated the onsets and offsets of vocalizations in Praat. Using a custom Python script (Table 1, Data file 1) [12, 13], I extracted three sorts of timing information: the duration of calls [14–17], the intervals between calls’ onsets [18, 19], and the intervals between calls’ maximum-intensity peaks [20].

**Table 1:** Overview of data files/data sets

Label	Name of data file/data set	File types (file extension)	Data repository and identifier (DOI or accession number)	License
*Data file 1*	*textgrid_IOI_IPI_dur_01*	*.py*	*Figshare* (10.6084/m9.figshare.5616490)	*CC*-*BY*
*Data file 2*	*durations_all*	*.csv*	*Figshare* (10.6084/m9.figshare.5616490)	*CC*-*BY*
*Data file 3*	*IOI_all*	*.csv*	*Figshare* (10.6084/m9.figshare.5616490)	*CC*-*BY*
*Data file 4*	*IPI_all*	*.csv*	*Figshare* (10.6084/m9.figshare.5616490)	*CC*-*BY*
*Data file 5*	*IOI_short*	*.csv*	*Figshare* (10.6084/m9.figshare.5616490)	*CC*-*BY*
*Data file 6*	*IPI_short*	*.csv*	*Figshare* (10.6084/m9.figshare.5616490)	*CC*-*BY*
*Data file 7*	*supplement_datanote01*	*.docx*	*Figshare* (10.6084/m9.figshare.5616490)	*CC*-*BY*

## Data description

### Subject

I recorded a female harbor seal pup. The seal was born in the wild and brought into rehabilitation at the Sealcentre Pieterburen, The Netherlands [21, 22], at the estimated age of 7 days [14, 22]. The animal was individually housed in a pool situated in a 1-room cabin. Seals in rehabilitation are usually housed in pairs [14]; this recording exploited the rare occurrence of individual housing.

### Sound recordings

On the twenty-first day from estimated birth, 10 min of vocalizations were recorded in air using a unidirectional microphone Sennheiser ME-66 (frequency response: 40–20,000 Hz ± 2.5 dB; Sennheiser electronic GmbH&Co. KG, Wedemark, Germany) [14]. The microphone was equipped with a MZW-66 foam windshield, and was connected to a digital recorder Zoom H6 (Zoom Corporation, Tokyo, Japan). Recordings, collected at 0.5–2 m distance from the seal, were saved as a .wav file (48 kHz sampling frequency; 24-bit quantization).

### Call annotations

The audio file was manually annotated in Praat version 6.0.11 [23]. Mother attraction calls (MACs) and other calls were annotated as two different categories on one tier. The tier was saved as a .TextGrid file. Only clear MACs [10, 11, 17, 24] were retained for further computations [14, 15, 17].

### Extraction of temporal variables

A Python 2.7 script extracted and combined annotations and sound features (Table 1, Data file 1), and outputted five .csv files. The script imported the annotations using package TextGridTools 1.4.3 [12] and the wave sound using Parselmouth [13]. The script calculated: durations (Table 1, Data file 2), inter-onset intervals (IOIs), and inter-peak intervals (IPIs) of calls. An IOI was defined as the time elapsed between the *onsets* of two consecutive calls (Table 1, Data file 3). An IPI was defined as the time between the maximum-intensity *peaks* of two consecutive calls (Table 1, Data file 4) [20]. Two more datasets were computed and output: short IOIs (IOI^s^, Table 1, Data file 5) and short IPIs (IPI^s^, Table 1, Data file 6), consisting of intervals within approximately 4 times the minimum value (≈ 3900 ms). The purpose of this threshold was to focus on timing *within* vocalization bouts (IOI^s^ and IPI^s^) as opposed to pooled timing within and *between* bouts (IOI and IPI).

### Descriptive statistics

Mean call duration was 976.1 ms (standard deviation **σ** = 205.7, see also Table 1, Data file 7). Mean IOI was 8578.3 ms (**σ** = 7807.4). Mean IPI was 8574.6 ms (**σ** = 7839.8). No significant difference was detected between these two distributions (Two-sample Kolmogorov–Smirnov test, D = 0.04, p = 0.99). Mean IOI^s^ was 1983.2 ms (**σ** = 722.1). Mean IPI^s^ was 2020.8 ms (**σ** = 803.3). No significant difference was detected between distributions of IOI^s^s and IPI^s^s (Two-sample Kolmogorov–Smirnov test, D = 0.10, p = 0.99). In other words, using onsets instead of peaks does not yield a significant difference between distributions. This holds at *two different timescales*, i.e. for both the IOI/IPI and the IOI^s^/IPI^s^ comparisons. The distributions of IOI and IPI have very high **σ**, almost equal to their means (CV, coefficient of variation, equals 0.91 for IOI and IPI). Conversely, the distributions of IOI^s^ and IPI^s^ have lower **σ** (CV equals 0.36 for IOI^s^ and 0.39 for IPI^s^).

## Limitations

A clear limitation of these data is their focus on one individual. Pups in rehabilitation are usually kept in groups. Hence, it is uncommon to record long runs of vocalizations from isolated individuals. Data from this individual seal served its purpose of tailoring an experiment to her [25]. However, solid inference about rhythm ontogeny, learning, individual differences, and species differences will require additional data [7]. It would be desirable to collect a panel dataset, where multiple animals are recorded daily, showing variance over individuals and time. Such dataset would enable comparing the (1) type of temporal distributions, (2) average length, and (3) degree of isochronous regularity, both between species (e.g. Phocids vs. Otariids [6, 7, 18]) and between housing conditions (lonely vs. in-pair housing). Seal pups’ call duration is known to vary with age [14, 15, 17]; all the other temporal variables presented in this Data Note are rarely investigated in pinniped bioacoustics.

A second limitation is that vocalizations were exclusively recorded in-air, while harbor seal pups [17] and adults [16] also vocalize underwater. This might not be an issue, because the medium of sound transmission should affect spectral, rather than temporal, properties of the calls [6]. However, vocal production repertoires might also vary across media, with some vocalizations only appearing in-air or underwater. Past research found that call *duration* is comparable across media [17]; comparison of *IOIs* and *IPIs* across media remains, to my knowledge, unexplored.

To comply with Data Note articles’ guidelines, this paper lacks analyses. Although simple descriptive statistics are suited for the original purpose of these recordings, namely estimating the mean IOI^s^ and using it to produce experimental stimuli, some analyses could be performed [19, 26].

## Abbreviations

MAC: mother attraction calls
IOI: inter-onset interval
IOI^s^: short inter-onset interval
IPI: inter-peak interval
IPI^s^: short inter-peak interval
σ: one standard deviation from the mean
CV: coefficient of variation
