# Correction to: Spontaneous rhythms in a harbor seal pup calls

**DOI:** 10.1186/s13104-018-3471-x

**Published:** 2018-06-11

**Authors:** Andrea Ravignani

**Affiliations:** 1 Research Department, Sealcentre Pieterburen, Hoofdstraat 94a, 9968 AG Pieterburen, The Netherlands; 2 Artificial Intelligence Lab, Vrije Universiteit Brussel, Pleinlaan 2, 1050 Brussels, Belgium; 3 Language and Cognition Department, Max Planck Institute for Psycholinguistics, Wundtlaan 1, 6525 XD Nijmegen, The Netherlands

## Correction to: BMC Res Notes (2018) 11:3 10.1186/s13104-017-3107-6

Following publication of the original article [1], the author reported an error in the first sentence of the ‘Sound recordings’ section. The recordings mentioned in this sentence were performed 6 days later than written. In this Correction article the original and corrected version of the sentence are presented.

The incorrect sentence upon publication:

- On the twenty-first day from estimated birth, 10 min of vocalizations were recorded in air using a unidirectional microphone Sennheiser ME-66 (frequency response 40–20,000 Hz ± 2.5 dB; Sennheiser electronic GmbH&Co. KG, Wedemark, Germany) [14].

The corrected sentence:

- On the twenty-seventh day from estimated birth, 10 min of vocalizations were recorded in air using a unidirectional microphone Sennheiser ME-66 (frequency response 40–20,000 Hz ± 2.5 dB; Sennheiser electronic GmbH&Co. KG, Wedemark, Germany) [14].

The reference which is cited in the above sentence can be reviewed in the original article.
